# The type 1 diabetes susceptibility locus *Idd5* favours robust neonatal development of highly autoreactive regulatory T cells in the NOD mouse

**DOI:** 10.3389/fimmu.2024.1358459

**Published:** 2024-02-09

**Authors:** Jérémy C. Santamaria, Sylvia Vuillier, Ariel O. Galindo-Albarrán, Sarah Castan, Claire Detraves, Olivier P. Joffre, Paola Romagnoli, Joost P. M. van Meerwijk

**Affiliations:** Toulouse Institute for Infectious and Inflammatory Diseases (Infinity), Institut National de la santé et de la recherche médicale (Inserm) UMR1291 – Centre national de la recherche scientifique (CNRS) UMR5051 – University Toulouse III, Toulouse, France

**Keywords:** thymus, regulatory T cells, immune tolerance, type 1 diabetes, Idd5

## Abstract

Regulatory T lymphocytes expressing the transcription factor Foxp3 (Tregs) play an important role in the prevention of autoimmune diseases and other immunopathologies. Aberrations in Treg-mediated immunosuppression are therefore thought to be involved in the development of autoimmune pathologies, but few have been documented. Recent reports indicated a central role for Tregs developing during the neonatal period in the prevention of autoimmune pathology. We therefore investigated the development of Tregs in neonatal NOD mice, an important animal model for autoimmune type 1 diabetes. Surprisingly, we found that, as compared with seven other commonly studied inbred mouse strains, in neonatal NOD mice, exceptionally large proportions of developing Tregs express high levels of GITR and PD-1. The latter phenotype was previously associated with high Treg autoreactivity in C57BL/6 mice, which we here confirm for NOD animals. The proportions of newly developing GITR^high^PD-1^+^ Tregs rapidly drop during the first week of age. A genome-wide genetic screen indicated the involvement of several diabetes susceptibility loci in this trait. Analysis of a congenic mouse strain confirmed that *Idd5* contributes to the genetic control of GITR^high^PD-1^+^ Treg development in neonates. Our data thus demonstrate an intriguing and paradoxical correlation between an idiosyncrasy in Treg development in NOD mice and their susceptibility to type 1 diabetes.

## Introduction

Regulatory T lymphocytes (“Tregs”) play a central role in the control of immune responses. The importance of these cells in the prevention of autoimmune and inflammatory pathologies is best illustrated by the observation that individuals and mice with mutations in the gene encoding FOXP3/Foxp3, the “master” regulator for Treg function, develop lethal autoimmune and inflammatory pathology, of which type I diabetes (“T1D”) is, in humans, a prominent component (1). In the etiology of T1D in individuals carrying polymorphisms in genes other than *FOXP3*, more subtle defects in the action of Tregs may be involved.

Most Tregs develop as an independent lineage in the thymus. In this organ, agonist recognition—by developing T cells—of MHC/self-peptide complexes drives the development of an autospecific Treg repertoire particularly adapted to the control of autoimmune responses (2). In mice, the Treg population emerging from the thymus is phenotypically and functionally diverse (3–5). Also, Tregs developing at distinct time points during life have distinct roles. Thus, Tregs developing in neonates protected substantially better from lethal autoimmune pathology than Tregs developing in adult animals (6). These observations urge for a more detailed analysis of Tregs when assessing their potential implication in the etiology of immunopathology.

Inherited defects in Treg-mediated immunoregulation may be involved in susceptibility to autoimmune diseases such as T1D. It was reported that the TCR repertoire expressed by Tregs developing in NOD mice was of limited diversity (7), but our recent high-throughput TCR-mRNA sequencing data challenged this conclusion (8). Polymorphisms in genes encoding IL-2 and its receptor components, which may affect *in-vivo* Treg function, are linked to diabetes in humans and mice (9–13). *In-vitro* Treg-mediated suppression of activation of T cells from NOD mice or T1D patients is of limited efficacy (14–16). Finally, polymorphisms in the *Idd9.1* and *Idd6* loci appear to affect *in-vivo* Treg function in NOD mice (17, 18). These data suggest that Treg-mediated suppression is defective in T1D in humans and mice. Further elucidation of the involved mechanisms and genetic polymorphisms should help the design of novel diagnostic and therapeutic tools.

Differentiation and selection of the T-cell repertoire in the thymus plays a central role in the *in-vivo* function of these cells. Therefore, we and others compared Treg development in the thymus of NOD mice to that in the T1D-resistant reference strain C57BL/6 (B6). Paradoxically, in NOD mice, substantially more Tregs develop than in B6 animals (19, 20). Our more recent work demonstrated that this phenomenon only occurs in neonatal mice. Later on, Tregs that had recirculated from the periphery back to the thymus inhibit the particularly robust Treg development in NOD mice, which thus reaches a level similar to that found in B6 animals. We showed that Treg recirculation was very prominent in NOD mice and that this was due to exceptionally strong Treg activation in peripheral lymphoid organs (21). Treg development in NOD vs. B6 mice therefore appears different which may be involved in the distinct susceptibility of these mice for T1D.

Based on the particularly robust activation of peripheral Tregs we previously reported in NOD mice, we here investigated the potential qualitative differences in Treg development, focusing on the very important neonatal Treg population.

## Results

### Particularly robust thymic production of GITR^high^PD-1^+^ Tregs in neonatal NOD mice

**Figure 1:**
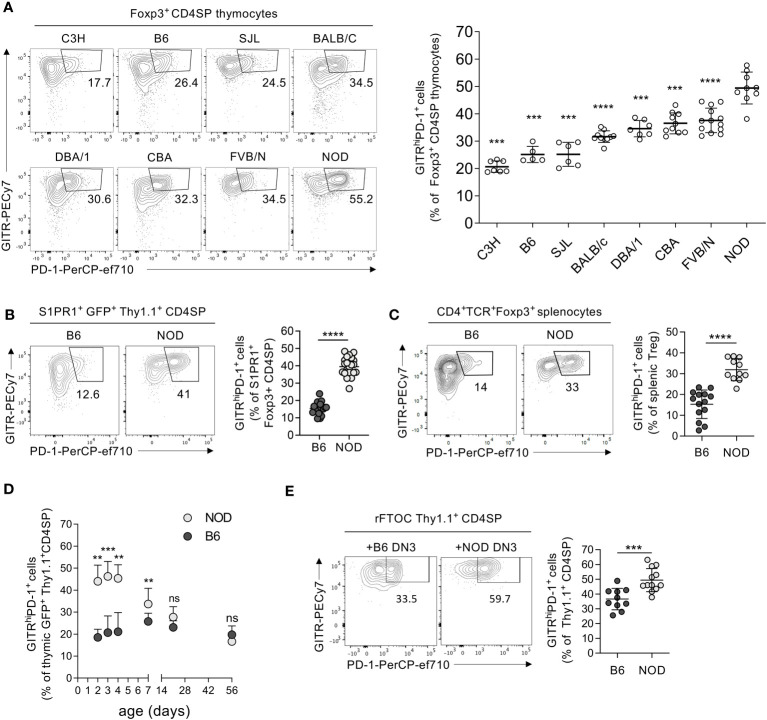
Particularly robust production of GITR^high^PD-1^+^ Tregs by the neonatal NOD thymus. **(A)** Thymocytes from 3-day-old mice of indicated strains were analyzed by flow cytometry using indicated markers (cf. **Supplementary Figure S1A**). The left panels show typical PD-1 vs. GITR expression patterns on CD4^+^CD8^−^TCR^+^ (CD4SP) Foxp3^+^ cells, and the right panels show the quantification using gates indicated in the left panel. **(B)** As in **(A)** but analyzing 4-day-old *Foxp3-Thy1^a^ Rag2-Gfp* mutant B6 vs. NOD mice and thymus exit-competent S1PR1^+^GFP^+^Thy1.1^+^ CD4SP thymocytes (cf. **Supplementary Figure S1C**). **(C)** As in **(B)** but analyzing CD4^+^TCR^+^Foxp3^+^ splenocytes (Tregs). **(D)** Kinetics of GITR^high^PD-1^+^ Treg development in *Foxp3-Thy1^a^ Rag2-Gfp* mutant B6 vs. NOD mice. **(E)** NMRI fetal thymi were depleted of hematopoietic cells before being reconstituted with adult *Foxp3-Thy1^a^ Rag2-Gfp* B6 or NOD DN3 precursors (cf. **Supplementary Figure S1D**). After 12 days of culture, the proportions of GITR^high^PD-1^+^ cells among developing CD4^+^CD8^−^TCR^−^Thy1.1^+^ Tregs were determined by flow cytometry. In **(A–C)**, dots represent individual mice and in **(E)** individual thymic lobes, and bars are mean values ± SD. In **(D)**, dots indicate mean values and bars SD. ns, not significant; **, *p* < 0.01; ***, *p* < 0.001; ****, *p* < 0.0001, Mann–Whitney test.

It was previously shown that Tregs developing during the neonatal period protect better from lethal autoimmunity upon adoptive transfer into AIRE-deficient NOD mice than cells differentiating in adult animals (6). We investigated the development of this population in NOD mice and compared it with that in other commonly used inbred mouse strains. Wyss and colleagues previously showed that newly developed Tregs expressing a GITR^high^PD-1^+^ phenotype are more autoreactive than their GITR^+^PD-1^−^ counterparts (3). We therefore investigated the GITR vs. PD-1 phenotypes of Tregs newly developing in neonates (3 to 4 days old) of B6, BALB/c, C3H, CBA, DBA/1, FVB, NOD, and SJL mice. The proportions of GITR^high^PD-1^+^ cells among CD4^+^CD8^−^TCR^high^Foxp3^+^ Tregs (gated as shown in **Supplementary Figure S1A**) varied dramatically, from 20.6% ± 2.1% in C3H mice to 49.4% ± 5.8% in NOD animals (**Figure 1A**). NOD mice also had particularly great proportions of GITR^high^PD-1^+^ Tregs among CD4^+^CD8^−^TCR^high^ thymocytes (CD4SP, **Supplementary Figure S1B**). These data suggested a genetic control of the development of strongly autoreactive GITR^high^PD-1^+^ Tregs, paradoxically very robust in the NOD mouse.

Treg precursors are not entirely resistant to thymic deletion (22). It was therefore important to evaluate if the potentially highly autoreactive newly developed GITR^high^PD-1^+^ Tregs can leave the thymus and populate the periphery. To assess this question, we first analyzed the expression of S1PR1, sufficient for the thymic export of T cells (23). Among thymus export-competent (S1PR1^+^) Tregs, we observed substantially more GITR^high^PD-1^+^ cells in NOD mice than in B6 neonates (**Figure 1B**, **Supplementary Figure S1C**). We also observed much higher proportions of GITR^high^PD-1^+^ cells among peripheral (splenic) Tregs in 4-day-old NOD than in age-matched B6 mice (**Figure 1C**).

Phenotypic differences between NOD and B6 Tregs have, to our knowledge, not been described. Moreover, it was reported that very autoreactive Tregs develop during the neonatal period, but not later on (24, 25). We therefore assessed the kinetics of GITR^high^PD-1^+^ Treg development in NOD and B6 mice. Intriguingly, we observed higher levels of these cells in NOD than in B6 thymi only during the first week of age. Already at 1 week of age, the proportion of developing GITR^high^PD-1^+^ Tregs had strongly dropped in NOD mice, and later on, they were similar in the two mouse strains (**Figure 1D**). Combined, these data indicate that, paradoxically, particularly high proportions of potentially strongly autoreactive GITR^high^PD-1^+^ Tregs develop during the neonatal period in the NOD thymus and populate peripheral lymphoid organs.

### The robust GITR^high^PD-1^+^ NOD Treg development is genetically controlled in a T-cell lineage-intrinsic manner

The difference in neonatal GITR^high^PD-1^+^ Treg development between NOD and B6 mice may be controlled by genetic factors acting within developing T cells and/or provided by the thymic microenvironment. To investigate this issue, we generated reconstituted fetal thymus organ cultures (rFTOC, **Supplementary Figure S1D**). We depleted fetal NMRI thymi of hematopoietic cells and we seeded these emptied thymic lobes with T-cell precursors from 8-week-old B6 or NOD mice. To avoid the potential contribution of dendritic cells and B lymphocytes developing from thymic T-cell precursors, we used FACS-sorted thymocytes at the DN3 stage of development (CD4^−^CD8^−^TCR^−^CD44^−^CD25^+^), known to only have T-cell precursor potential (26). Whereas 36.6% ± 7.2% of Tregs developing from B6 DN3 in such rFTOC expressed PD-1 and high levels of GITR, 49.4% ± 7.8% of NOD Tregs had this phenotype (**Figure 1E**). These results indicated that part of the difference in GITR^high^PD-1^+^ Treg development between B6 and NOD mice was due to factors acting within the T-cell compartment. Since the precursor cells seeded into the cultures were of adult origin, our observations also showed that the difference is not caused by factors intrinsic to newborn precursors.

### GITR^high^PD-1^+^ NOD Tregs have a phenotype suggesting high autoreactivity

**Figure 2:**
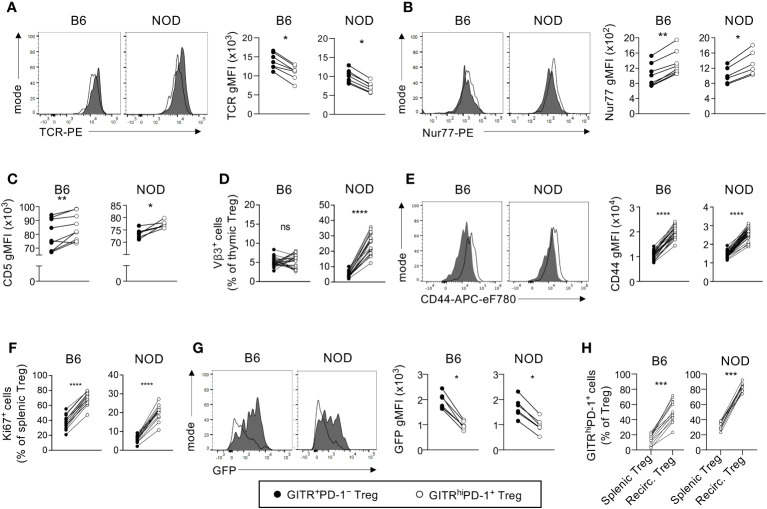
Neonatal GITR^high^PD-1^+^ Tregs are more autoreactive than their GITR^+^PD-1− counterparts. **(A, B)** Thymocytes from indicated 3-day-old *Foxp3-Thy1^a^* B6 and NOD neonates were analyzed by flow cytometry. Depicted are typical patterns (left panels) and quantifications (gMFI, right panels) of the expression of indicated markers by CD4^+^CD8^−^Thy1.1^+^ cells (Tregs) (gated as shown in **Supplementary Figure S2A**). **(C)** As in **(A, B)** but indicating the mean CD5 expression levels. **(D)** The proportions of Mtv-3 superantigen-specific cells among GFP^+^CD4^+^CD8^−^Thy1.1^+^ Tregs in 4-day-old *Foxp3-Thy1^a^* Mtv-3^−^ B6 and Mtv-3^+^ NOD mice. **(E–G)** As in (**A–D**) but for CD4^+^Thy1.1^+^ splenocytes (Tregs, gated as shown in **Supplementary Figure S2D**) from 4-day-old neonates. Typical flow cytometry patterns for data shown in **(C, D, F)** are shown in **Supplementary Figures S2B, C, E**. **(H)** GITR^high^PD-1^+^ preferentially recirculate back to the thymus. The proportions of GITR^high^PD-1^+^ cells among peripheral Tregs (splenic Tregs) and among recirculating GFP^−^ Tregs (Recirc. Tregs) in the thymus of 4-day-old B6 and NOD mice. Dots represent individual mice and lines represent paired data (same mouse). *, *p* < 0.05; **, *p* < 0.01; ***, *p* < 0.001; ****, *p* < 0.0001, Wilcoxon test.

It was paradoxical to find particularly robust development of GITR^high^PD-1^+^ and, therefore, presumably highly autoreactive, neonatal Tregs in the T1D-prone NOD mouse. However, the highly autoreactive nature of GITR^high^PD-1^+^ Tregs was described for B6 mice (3) and may or not be valid for NOD Tregs. Also, for technical reasons, we did not include in our study the CD25 marker used by Wyss and colleagues, which might bias the interpretation of our results. We therefore compared the autoreactivity of GITR^high^PD-1^+^ vs. GITR^+^PD-1^–^ Tregs in NOD and B6 mice. Neonatal GITR^high^PD-1^+^ Tregs from B6 and NOD mice expressed significantly lower levels of TCR and higher levels of Nur77 and CD5 than GITR^+^PD-1^–^ cells (**Figures 2A–C**, **Supplementary Figures S2A, B**). These data suggested a similarly higher autoreactivity of GITR^high^PD-1^+^ than of GITR^+^PD-1^–^ Tregs in NOD and B6 mice (27–29), thus confirming and extending previously published data on B6 animals (3). Moreover, in NOD but not in B6 mice, thymic stromal cells express a superantigen encoded by the endogenous mammary tumor virus-3 (Mtv-3). When presented by the MHC class II molecule I-A^g7^, the Mtv-3 superantigen strongly activates all T cells expressing the TCR Vβ3 variable segment. Strongly autoreactive Vβ3-expressing T-cell precursors are therefore deleted in the NOD thymus (30). Given that superantigens can also induce the selection of reactive Tregs (31), we investigated the development of Vβ3-expressing Tregs, highly autoreactive in Mtv-3-expressing NOD but not in B6 mice which lack Mtv-3. We found many more Vβ3-expressing cells among GITR^high^PD-1^+^ than among GITR^+^PD-1^–^ Tregs in NOD but not in B6 neonates (**Figure 2D**, **Supplementary Figure S2C**). Combined, these data suggested that, as in B6 mice, in NOD animals GITR^high^PD-1^+^ Tregs were substantially more autoreactive than GITR^+^PD-1^–^ cells.

### GITR^high^PD-1^+^ NOD Tregs are highly reactive *in vivo*

To formally assess the particularly (most probably auto-) reactive nature of NOD GITR^high^PD-1^+^ Tregs, we next analyzed their activation in the periphery. Upon leaving the thymus, a large proportion of Tregs is activated in the periphery (32). The expression levels of the T-cell activation marker CD44 were noticeably higher on GITR^high^PD-1^+^ than on GITR^+^PD-1^–^ Tregs (**Figure 2E**, **Supplementary Figure S2D**). We also found many more cells expressing the proliferation marker Ki67 among GITR^high^PD-1^+^ than among GITR^+^PD-1^–^ Tregs (**Figure 2F**, **Supplementary Figure S2E**). In *Rag2-Gfp* mice, GFP accumulates in developing T cells up to the extinction of *Rag2* expression upon positive selection and then degrades with an *in-vivo* half-life of 56 h (33). The proliferation of recent thymic emigrants, which still express detectable levels of GFP, will lead to the dilution of GFP. At the age of 4 days, Tregs just start to leave the mouse thymus (34). In the spleen of 4-day-old mice, GITR^high^PD-1^+^ recent thymic emigrant Tregs displayed markedly lower GFP fluorescence levels than GITR^+^PD-1^–^ cells (**Figure 2G**), indicating a higher proliferation rate of GITR^+^PD-1^+^ cells compared with their GITR^+^PD-1^–^ counterparts. Upon their activation in the periphery, Tregs recirculate back to the thymus (21, 35). If GITR^high^PD-1^+^ are more activated than GITR^+^PD-1^–^ cells, the former cells should preferentially recirculate to the thymus. Consistent with this premise, we found that the proportions of GITR^high^PD-1^+^ cells among recirculating GFP^−^ Tregs in the thymus were much higher than those among Tregs in the spleen of B6 newborns and, to an even greater extent, of NOD mice (**Figure 2H**). These data indicate that GITR^high^PD-1^+^ Tregs preferentially recirculate back to the thymus. Combined, our data confirm and extend the particularly (probably auto-) reactive nature of GITR^high^PD-1^+^ Tregs (3). Importantly, we obtained very similar differences between GITR^high^PD-1^+^ vs. GITR^+^PD-1^–^ Tregs in B6 and NOD mice. Taken together, these data further indicate that a particularly high proportion of strongly autoreactive Tregs develops in the neonatal NOD thymus.

### At least two genomic loci control development of GITR^high^PD-1^+^ Tregs

**Figure 3:**
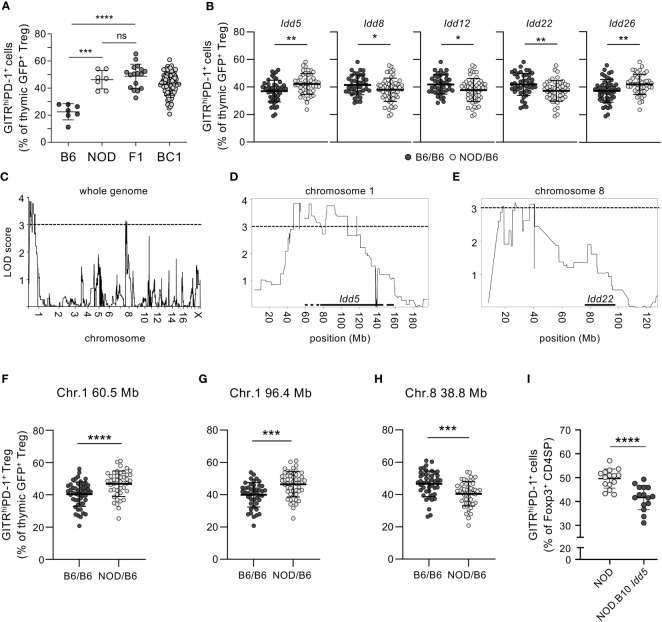
Genetic control of neonatal development of GITR^high^PD-1^+^ Tregs. **(A)** Thymocytes from indicated 3-day-old *Rag2-Gfp Foxp3-Thy1^a^*-mutant neonatal mice were analyzed by flow cytometry and the proportions of GITR^high^PD-1^+^ cells among GFP^+^CD4^+^CD8^−^TCR^+^Thy1.1^+^ cells (Tregs) determined. F1, (NODxB6)F1; BC1, (F1xB6)BC1. **(B)** Proportions of GITR^high^PD-1^+^ cells among thymic Tregs in BC1 mice carrying B6 and/or NOD alleles of indicated *Idd* (as determined by PCR). For the complete list of analyzed *Idd*, see **Supplementary Figure S4**. **(C–H)** Genome-wide SNP profiling was performed on genomic DNA from B6, NOD, and 94 BC1 mice (f/m). LOD scores of GITR^high^PD-1^+^ Treg development for **(C)** the whole genome (except for the Y-chromosome), **(D)** chromosome 1, and **(E)** chromosome 8. Locations of *Idd5* and *Idd22* are indicated with horizontal bars. The proportions of GITR^high^PD-1^+^ cells among Tregs in BC1 mice carrying the indicated B6 and/or NOD alleles on **(F)** Chr. 1, 60.5 Mb; **(G)** Chr. 1, 96.4 Mb; **(H)** Chr. 8, 38.8 Mb; and **(I)** in 4-day-old NOD vs. NOD.B10 *Idd5* congenic mice. Dots represent individual mice, and bars mean values ± SD. ns, not significant; *, *p* < 0.05; **, *p* < 0.01; ***, *p* < 0.001; ****, *p* < 0.0001, Mann–Whitney test. In **(C–E)**, the horizontal dashed lines indicate the LOD score of 3.0 above which the genetic linkage was considered statistically significant.

The very diverse levels of GITR^high^PD-1^+^ Tregs we found in distinct inbred mouse strains (**Figure 1A**) suggested that their development is modulated by genetic factors. To more formally address this possibility, we next performed a genetic analysis. We crossed NOD mice to B6 animals and analyzed neonatal Treg development. In the thymus of F1 mice, we found proportions of GITR^high^PD-1^+^ cells among Tregs similar to those observed in NOD mice (**Figure 3A**). The B6 phenotype therefore appears recessive. We then backcrossed F1 mice to B6 animals. These “backcross 1” (BC1) animals displayed a large diversity in the proportions of GITR^high^PD-1^+^ cells among Tregs (**Figure 3A**). These results formally confirmed genetic control of the development of GITR^high^PD-1^+^ Tregs and indicated its polygenic nature.

The I-A^g7^ allele expressed by NOD mice is a major T1D susceptibility locus (“*Idd1*”) and regulates non-cognate negative selection in the thymus (36). Given the highly autoreactive nature of GITR^high^PD-1^+^ Tregs, we postulated that I-A^g7^ may play an important role in their differentiation. To assess this possibility, we identified homozygous I-A^b/b^ and heterozygous I-A^b/g7^ mice among the BC1 neonates and compared the proportions of GITR^high^PD-1^+^ cells among newly developing (i.e., GFP^+^) Tregs. We did not observe any difference between I-A^b/b^ and heterozygous I-A^b/g7^ mice (**Supplementary Figure S3**), excluding a role for the I-A^g7^ allotype in GITR^high^PD-1^+^ Treg development.

More than 40 other T1D susceptibility loci have been identified (37). To investigate the potential implication of these loci in the development of GITR^high^PD-1^+^ Tregs, we first determined the *Idd* genotypes of the BC1 neonates by PCR and then compared the proportions of GITR^high^PD-1^+^ Tregs among GFP^+^ Thy1.1^+^ CD4SP Tregs (**Supplementary Figure S4**). Several *Idd* appeared involved in the development of these cells. Thus, the heterozygous presence of the NOD alleles of *Idd5* and *Idd26* (both localized on chromosome 1, “Chr. 1”) conferred increased proportions of GITR^high^PD-1^+^ Tregs (**Figure 3B**). By contrast, the heterozygous presence of the NOD allele of *Idd8* (Chr. 14), *Idd12* (Chr. 14), and *Idd22* (Chr. 8) unexpectedly conferred reduced proportions of GITR^high^PD-1^+^ Tregs (**Figure 3B**). These data indicate that T1D susceptibility loci genetically control the development of GITR^high^PD-1^+^ Tregs.

To comprehensively identify genomic loci controlling the development of GITR^high^PD-1^+^ Tregs, we submitted genomic DNA from the BC1 animals to genome-wide, high-density single nucleotide polymorphism (SNP) profiling (**Figure 3C**). We thus identified loci on chromosomes (Chr.) 1 and 8 that control the difference in GITR^high^PD-1^+^ Treg development between B6 and NOD mice (**Figures 3D, E**). On Chr. 1, two loci may be involved (**Figure 3D**). In BC1 mice heterozygous (B6/NOD) for these two loci, significantly more GITR^high^PD-1^+^ Tregs developed than in homozygous (B6/B6) pups (**Figures 3F, G**). These observations confirm the dominance of the NOD allele(s) which favour(s) the development of GITR^high^PD-1^+^ Tregs. However, the difference between mice homozygous and heterozygous for these loci was substantially smaller than the difference between B6 and (B6xNOD)F1 mice (cf. **Figures 3A, F, G**), again indicating the polygenic control of the GITR^high^PD-1^+^ Treg phenotype. In BC1 neonates heterozygous (B6/NOD) for the locus identified on Chr. 8, significantly less GITR^high^PD-1^+^ Tregs developed than in homozygous (B6/B6) pups (**Figure 3H**). This observation indicates the dominance of the NOD allele(s) which, in contrast to the locus on Chr. 1, inhibits the development of GITR^high^PD-1^+^ Tregs. The NOD alleles of the loci on Chr. 1 and 8 therefore control GITR^high^PD-1^+^ Treg development in an opposite fashion.

### The type 1 diabetes susceptibility locus *Idd5* controls the development of GITR^high^PD-1^+^ Tregs

The identified loci on chromosome 1 largely overlap with the diabetes susceptibility locus *Idd5* (38) (**Figure 3D**). To assess the potential implication of *Idd5* in neonatal GITR^high^PD-1^+^ Treg development, we analyzed congenic NOD neonates carrying the *Idd5* locus of B10 origin (NOD.B10 *Idd5* mice, strain R974 (39)). Among Foxp3^+^ CD4SP Tregs in the thymi of NOD.B10 *Idd5* mice, we found lower proportions of GITR^high^PD-1^+^ cells than among NOD Tregs (**Figure 3I**). These data thus show that the particularly robust development of GITR^high^PD-1^+^ Tregs in NOD mice is, in part, controlled by the T1D susceptibility locus *Idd5*.

## Discussion

The data presented here indicate that, paradoxically, a particularly high proportion of Tregs developing in the thymus of neonatal NOD mice has the GITR^high^PD-1^+^ phenotype previously associated with high autoreactivity in B6 mice (3). We confirm that these Tregs are highly autoreactive in NOD animals. We found that robust GITR^high^PD-1^+^ Treg development was restricted to the neonatal period and rapidly dropped by 1 week of age when we did not observe a drop in GITR^high^PD-1^+^ Treg development in B6 mice. Genetic analyses indicated the involvement of diabetes susceptibility loci, in particular *Idd5*, suggesting a causative link with the disease. Our data thus suggest an indirect link between an idiosyncrasy in the development of Tregs in neonatal NOD mice and their susceptibility to T1D.

The Treg population developing in neonatal NOD mice appears substantially more autoreactive than that differentiating in the thymus of several other inbred mouse strains. This conclusion is based in part on the particularly high proportion of NOD Tregs expressing a GITR^high^PD-1^+^ phenotype, previously shown to be highly autoreactive in B6 mice (3). In the NOD as well as in the B6 thymus, GITR^high^PD-1^+^ Tregs expressed higher levels of Nur77 and CD5 and lower levels of TCR than GITR^+^PD-1^–^ cells, indicating recent stronger signaling through the TCR. Dong and colleagues recently found that intrathymic NOD vs. B6 Tregs expressed similar levels of CD5, but they analyzed adult animals and did not distinguish between GITR^+^PD-1^–^ and GITR^high^PD-1^+^ cells (40). In NOD mice, Mtv-3 superantigen-specific TCR Vβ3-expressing Tregs were enriched in the GITR^high^PD-1^+^ as compared with the GITR^+^PD-1^–^ Treg subset. In the periphery, the expression of the activation marker CD44 and of the proliferation marker Ki67 was substantially greater on GITR^high^PD-1^+^ than on GITR^+^PD-1^–^ Tregs, both in B6 and NOD mice, confirming higher reactivity, most probably to self-antigens. GITR^high^PD-1^+^ Tregs were also more represented among intrathymic Tregs that had recirculated back from the periphery than among peripheral Tregs, consistent with their higher *in-vivo* activation (21). Combined, these observations confirm the very autoreactive nature of GITR^high^PD-1^+^ Tregs in both mouse strains.

In the spleen, substantially more GITR^high^PD-1^+^ than GITR^+^PD-1^–^ Tregs proliferated in both NOD and B6 mice. However, for both populations, much fewer cells proliferated in NOD than in B6 mice. It was recently shown that whereas the diversity of the TCR repertoire expressed by newly developed Tregs is similar in B6 and NOD mice (8), in peripheral lymphoid organs, this diversity is higher in NOD than in B6 mice. This phenomenon was apparently due to reduced clonal Treg expansion in NOD mice (41), which in turn is probably caused by limited production of IL-2 in NOD as compared with B6 mice (9). It is therefore important to distinguish between the autoreactivity of Treg (controlled by their specificity and/or by other Treg-intrinsic factors) and their proliferation (controlled by, e.g., IL-2 availability or responsiveness).

Further work will be required to identify molecular mechanisms involved in the development of the particularly autoreactive nature of neonatal NOD Tregs. Several genes within the thus far identified genomic region encode products that are involved in Treg development and/or function: CTLA-4 modulates the selection of the TCR repertoire expressed by developing Tregs and plays a major role in the suppressive function of this T-cell population (42, 43), and Tregs appear to be functionally impaired in ICOS-deficient NOD mice (17). However, hundreds of other genes are encoded within the *Idd5* locus and may be involved (39). It will now be important to assess which gene(s) within the *Idd5* locus is (are) responsible for the development of the particularly autoreactive Treg population in the neonatal NOD thymus and which mechanisms are involved.

We observed that the particularly robust development of GITR^high^PD-1^+^ Tregs in the NOD mouse only occurs very early after birth and drops rapidly after. In rFTOC, we found that adult NOD precursors more efficiently develop into GITR^high^PD-1^+^ Tregs than adult B6 precursors, indicating that the decline we observed was not due to potential differences between neonatal and adult precursor cells. Our observation is coherent with the report that Tregs developing early in life are more autoreactive than Tregs developing later on (24). A potential explanation for the latter phenomenon lies in defective intrathymic deletion of autoreactive T-cell precursors early in life, allowing Tregs specific for certain autoantigens to differentiate (25). After the neonatal period, peripheral DC homing to the thymus induced the deletion of the very autoreactive T-cell precursors, pre-empting their differentiation into Tregs. However, the drop in the development of GITR^high^PD-1^+^ Tregs we observed in NOD mice occurred earlier than the drop in the development of Tregs specific for the autoantigens in B6 mice reported by Stadinski et al. (25). An alternative mechanism appears therefore involved. Rapid homing back to the thymus of Tregs activated in the periphery may somehow play a role (21).

Our genetic analyses indicate a link between a particularity in Tregs neonatally developing in NOD mice and their susceptibility to T1D. Since neonatal Tregs apparently play a crucial role in the protection from autoimmune pathology (6), we hypothesize that anomalies in these neonatal Tregs may somehow be involved in the etiology of T1D in the NOD mouse. For example, the expression of PD-1, a modulator of Treg activity (44–46), may hamper the capacity of Tregs to protect from T1D. The correlation between robust neonatal Treg autoreactivity and T1D susceptibility may also be related to the increased recirculation of activated peripheral Tregs back to the thymus which we observed. In the thymus, these cells may modulate the selection of Tconv, thus affecting their diabetogenic potential (35, 47, 48). It also remains possible that molecular mechanisms involved in robust GITR^high^PD-1^+^ Treg development operate in other cells and alter susceptibility to T1D in a Treg-independent manner. Finally, we cannot exclude the possibility that the idiosyncrasy in Treg development we here described is unrelated to T1D susceptibility. It will now be important to identify the precise gene(s) and mechanism involved in neonatal GITR^high^PD-1^+^ Treg development and to assess if and how it affects T1D susceptibility.

The observations described here may ultimately lead to the identification of genes and mechanisms involved in disease susceptibility of the NOD mouse, a very widely used animal model for T1D (49). This is an important issue since, despite the identification of more than 40 T1D susceptibility loci in mice and humans, only very few involved genes and mechanisms have thus far been identified (49). Understanding the potential defects in Treg-mediated immune suppression involved in susceptibility to T1D will also help in the development of innovative therapies against this debilitating disease affecting increasing numbers of children at a progressively younger age (50).

## Methods

### Mice

B6, BALB/c, C3H, CBA, DBA/1, and FVB mice were purchased from Janvier labs (Le Genest-Saint-Isle, FRA), and NOD and SJL were from Charles River (Wilmington, USA) Laboratories. *Foxp3-Thy1^a^ Rag2-Gfp*-mutant B6 and NOD mice were previously described (21). NOD.B10 *Idd5* congenic R974 mice (39) were from JAX (Bar Harbor, USA) Laboratories. All experiments involving animals were performed in compliance with governmental and institutional guidelines (ethical approval APAFIS#4151-201602171 0481496.v6).

### Antibodies

We used the following monoclonal antibodies (mAbs) and secondary reagents: APC-Cy7- and Pacific Blue-labeled anti-CD4 (GK1.5), biotin-labeled anti-CD4 (RM4.4), PE-CF594-labeled anti-CD8α (53.6.7), BV510-labeled anti-CD8β (H35-17.2), PE-Cy7- and PE-labeled anti-CD25 (PC61), PECF-BV421- and biotin-labeled anti-TCRβ (H57-597), BV421- and APC-labeled anti-Thy1.1 (OX-7), AF647-labeled anti-Ki67 (B56), PE-labeled anti-Nur77 (12.14), PE-labeled anti-CD5 (53-7.3), PE-labeled anti-I-Ab (AF6-120.1), biotin-labeled anti-RT1B (OX6) for the detection of I-Ag7, biotin-labeled anti-TCR Vβ3 antibody (KJ25) (all from BD (Franklin Lakes, USA) Biosciences); PECy7-labeled anti-GITR (DTA-1), PerCP-ef710-labeled anti-PD-1 (J43), biotin-labeled anti-Thy1.1 (HIS51), PE-labeled anti-TCRβ (H57-597), PerCP-Cy5.5-labeled or APC-ef780-labeled anti-CD44 (IM7), APC-labeled anti-CD62L (MEL-14), ef450-BV605-PE-Cy7- or PE-labeled streptavidin, PE-labeled anti-H-2K^d^ (SF1-1.1), biotin-labeled anti-H-2K^b^ (AF6-88.5), PE-labeled anti-CCR7 (4B12), ef450- and ef660-labeled anti-Foxp3 (FJK16S) (all from eBioscience (thermofisher) (Waltham, USA)); and BV605-labeled anti-CD73 (TY/11.8) (from Biolegend (San Diego, USA)); anti-S1P1/EDG-1 antibody (713412, R&D (Minneapolis, USA) Systems); and biotin-SP (long spacer) AffiniPure F(ab’)_2_ Fragment Donkey Anti-Rat IgG (H+L) (polyclonal, Jackson immuinoresearch (West Grove, USA)).

### Flow cytometry

Sample preparation and staining were essentially performed as previously described (51). FACS data were acquired using an LSRII or a Fortessa flow cytometer (BD Biosciences, San Jose, CA, USA) and analyzed using FlowJo software (Tree Star, Ashland, OR, USA). Doublets and dead cells were excluded from the analysis by using appropriate FSC/SSC gates.

### Reconstituted fetal thymus organ cultures

Thymic lobes from NMRI mice were collected and individually cultured on transwell inserts (0.4μm pores, polycarbonate membrane, Corning (Corning, USA)) at the air–liquid interface using a complete medium (10% FBS) containing 1.35 mM of 2-deoxyguanosine (2dGUO, Sigma (Burlington, USA)). Six days later, lobes were intensely washed and cultured for 24 h in hanging drops containing 50,000 DN3 (CD4^−^CD8^−^TCR^−^CD25^+^CD44^-^) cells FACS-sorted from thymocytes (CD8-depleted using mAb 31M and rabbit complement) from 8-week-old NOD or B6 mice. Lobes were then cultured on transwells for 12 days (cf. **Supplementary Figure S1D**). Lobes were collected and digested in RPMI containing 4 μg/ml of Liberase and 0.1 μg/ml of DNAse I (Roche (Basel, Switzerland)) for 5 min at 37°C with vigorous pipetting every 2 min. Cells were then washed, counted, and stained for flow cytometry analysis.

### *Idd* genotyping

DNA was prepared from tail clips of 3-day-old NOD, B6, F1, and BC1 mice using standard procedures. *Idd* genotyping was performed by PCR using published primers (52).

### Genome-wide SNP profiling

DNA was prepared from the tail clips of 3-day-old NOD, B6, and BC1 (*n* = 94) mice using standard procedures, quality-controlled using a fragment analyzer Agilent (Santa Clara, USA)), quantified using PicoGreen, amplified, and hybridized to Illumina GGP Mouse GIGA-MUGA Arrays according to the supplier’s instructions (Infinium HTS Assay Protocol Guide, 15045738 Rev.A October 2013, Manual Protocol). Arrays were scanned using an iScan System (Illumina (San Diego, USA)).

Thus, the obtained raw data were quality-controlled and analyzed using GenomeStudio Software 2010 with the Genotyping module (Illumina). After optimization of the cluster file and exclusion of uninterpretable SNPs, we retained 34,003 SNPs polymorphic between NOD and B6 (out of the 143,446 SNPs on the arrays). LOD scores were calculated and genome regions graphed with a customized script under R programming language using the “qtl2” package (53). The analysis was performed using the “backcross” parameter taking as inputs the genomic map, the physical map (mm10), and the phenotype table (% of GITR^high^PD1^+^ cells among thymic CD4^+^CD8^−^TCR^high^GFP^+^Thy1.1^+^ Tregs). The “error probabilities” parameter was arbitrarily fixed to 0.002. The script and tables are available at https://github.com/arielgalindoalbarran/Idd5NOD.

## Data availability statement

The original codes presented in the study are publicly available. This data can be found here: https://github.com/arielgalindoalbarran/Idd5NOD. The datasets presented in this study can be found in online repositories. The names of the repository/repositories and accession number(s) can be found below: GSE253484 (GEO).

## Ethics statement

The animal study was approved by comité d’éthique en expérimentation animale n°122. All experiments involving animals were performed in compliance with governmental and institutional guidelines (ethical approval APAFIS#4151-201602171 0481496.v6). The study was conducted in accordance with the local legislation and institutional requirements.

## Author contributions

JS: Formal analysis, Investigation, Methodology, Supervision, Writing – review & editing. SV: Formal analysis, Investigation, Methodology, Supervision, Writing – review & editing. AG-A: Formal analysis, Investigation, Methodology, Writing – review & editing. SC: Conceptualization, Formal analysis, Methodology, Writing – review & editing. CD: Formal analysis, Investigation, Methodology, Writing – review & editing. OJ: Conceptualization, Writing – review & editing. PR: Conceptualization, Formal analysis, Funding acquisition, Methodology, Supervision, Validation, Writing – review & editing. JvM: Conceptualization, Funding acquisition, Methodology, Project administration, Supervision, Validation, Writing – original draft, Writing – review & editing.
