# Supplementary material for: The type 1 diabetes susceptibility locus Idd5 favours robust neonatal development of highly autoreactive regulatory T cells in the NOD mouse
Source: Front Immunol. 2024 Feb 9;15:1358459. doi: 10.3389/fimmu.2024.1358459 (PMC10884962; doi:10.3389/fimmu.2024.1358459)
Supplement: Supplementary file 1 [file Presentation_1.pdf]

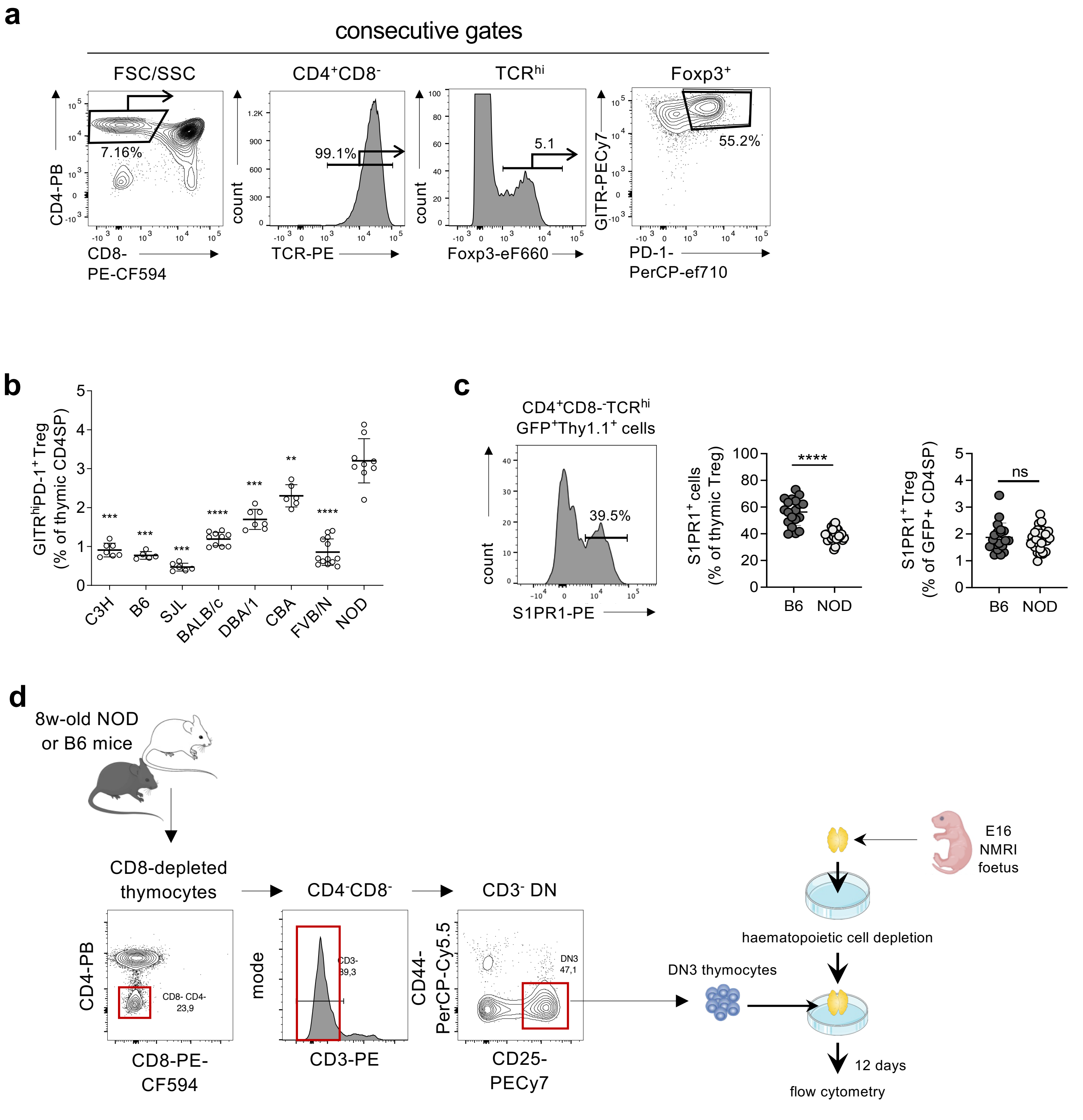

Figure S1. GITR/PD-1 and S1PR1-phenotypes of Treg developing in the thymi of distinct mouse strains

Thymocytes from neonates were analysed by flow-cytometry. **(a)** Gating strategy used to identify (FSC/SSC-gated) live $CD4^{+}CD8^{-}TCR^{high}Foxp3^{+}$ Treg and $GITR^{high}PD\text{-}1^{+}$ cells (NOD mouse in depicted example). Boxes/lines indicate subsequent $CD4^{+}CD8^{-}$, TCR^{hi}, $Foxp3^{+}$, and $GITR^{high}PD\text{-}1^{+}$ gates. **(b)** Proportions of $GITR^{high}PD\text{-}1^{+}$ Treg among $CD4^{+}CD8^{-}TCR^{hi}$ (CD4SP) thymocytes in three-day-old neonates of indicated mouse strains. **(c)** S1PR1-expression on four-day-old neonatal $CD4^{+}CD8^{-}TCR^{hi}Thy1.1^{+}GFP^{+}$ thymic Treg (left panel, *Foxp3-Thy1^a^ mutant* NOD mouse) and proportions of thymus egress-competent $S1PR1^{+}$ Treg. **(d)** To generate rFTOC, NMRI foetal thymi were depleted of haematopoietic cells before being reconstituted with *Foxp3-Thy1*^{a} DN3-precursors FACS-sorted from CD8-depleted B6 or NOD thymocytes. Red boxes indicate gates used for sorting. Dots represent individual mice, bars mean values ± SD. ns, not significant; **, p<0.01; ***, p<0.001; ****, p<0.0001, Mann-Whitney test.

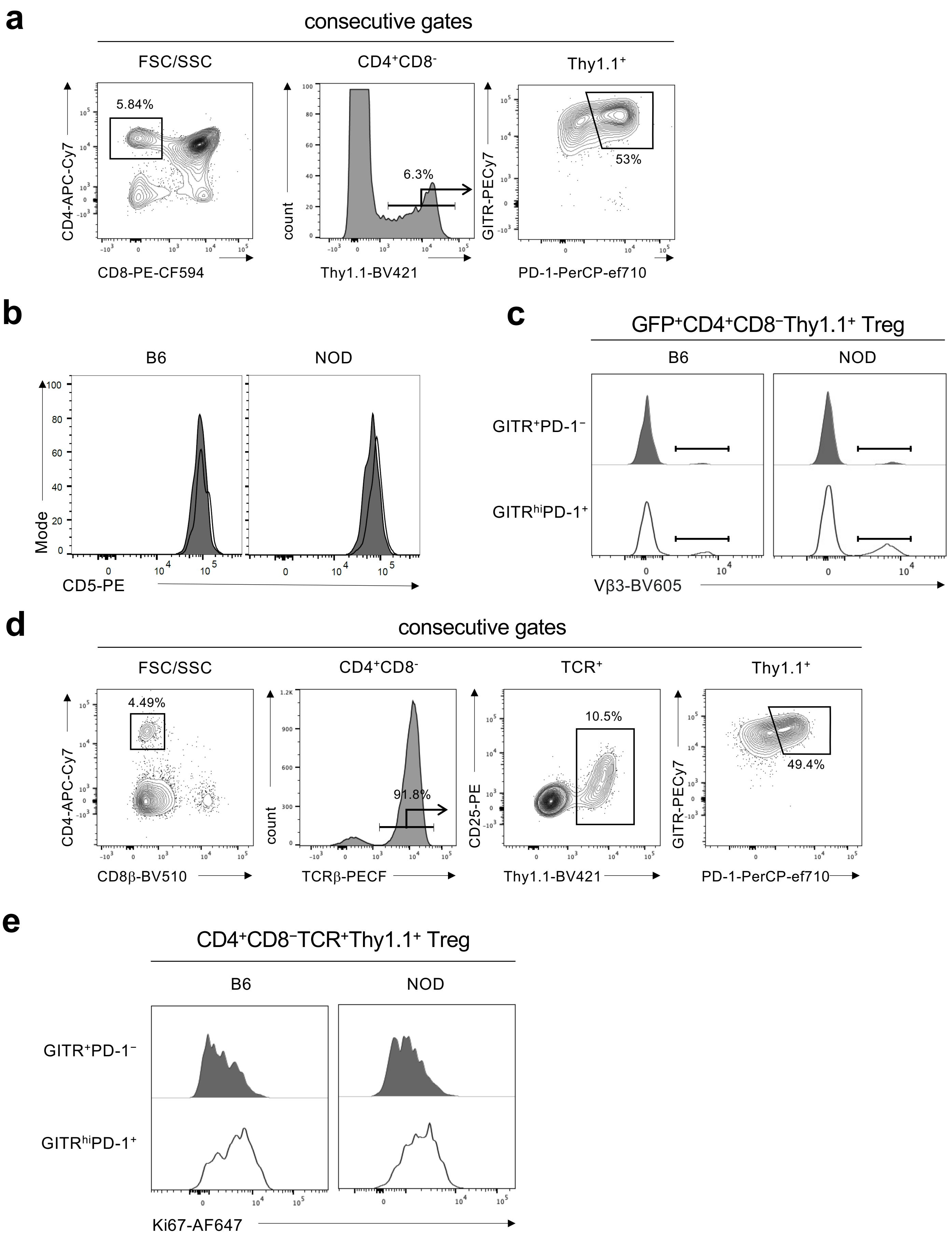

Figure S2. The $GITR^{high}PD\text{-}1^{+}$ Treg population in neonatal NOD thymi is enriched in autoreactive cells

(a-c) Thymocytes and **(d,e)** splenocytes from three- or four-day-old *Foxp3-Thy1^{a}* NOD neonates, respectively, were analysed by flow-cytometry using indicated markers and gates. Depicted are typical patterns of the expression of **(b)** CD5 and **(c)** Vβ3 by intrathymic $CD4^{+}CD8^{-}Thy1.1^{+}$ Treg and **(e)** Ki67 by splenic $CD4^{+}TCR^{+}Thy1.1^{+}$ Treg. For quantifications, see Fig. 2c, d, and f.

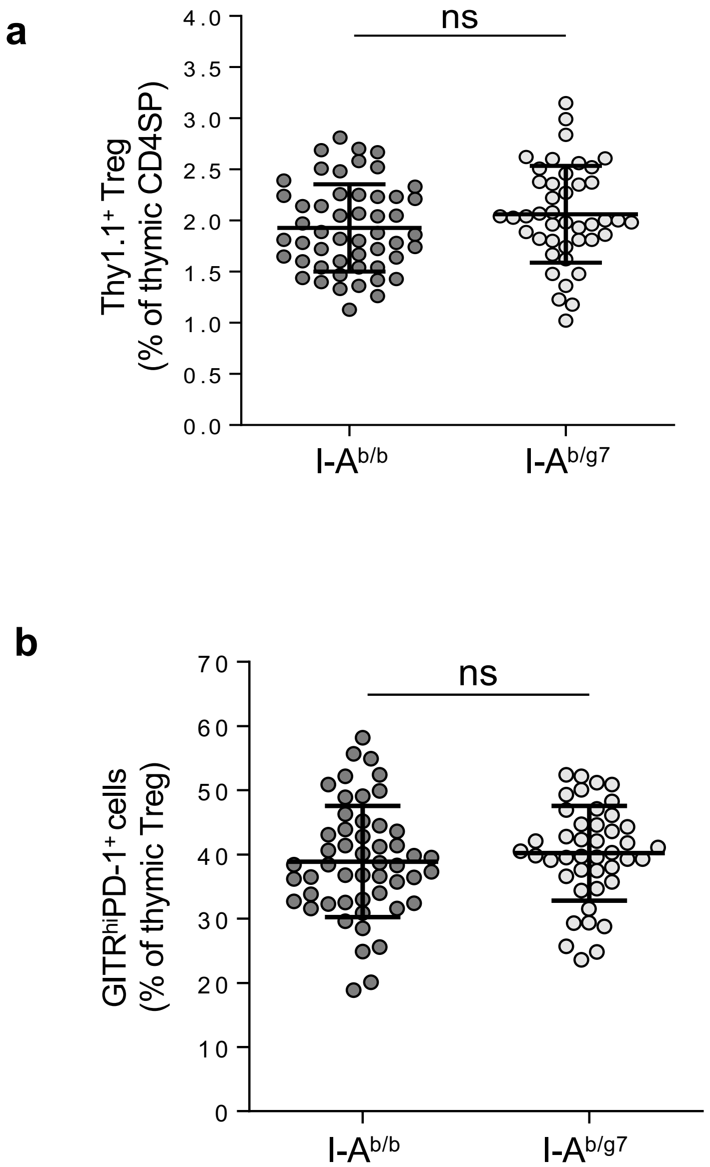

Figure S3. The MHC locus does not control GITR^{high}PD-1^{+} Treg development in neonatal BC1 mice

Thymocytes from three-day-old (F1xB6)$_{BC1}$ *Rag2-Gfp Foxp3-Thy1^{a}*-mutant neonates were analysed by flow-cytometry. I-A^{b} *vs*. I-A^{g7} genotypes/phenotypes were determined by flow cytometry. (**a**) Proportions of Thy1.1^{+} cells (Treg) among GFP^{+}CD4^{+}CD8^{-}TCR^{+} thymocytes (CD4SP). (**b**) Proportions of GITR^{high}PD-1^{+} cells among GFP^{+}CD4^{+}CD8^{-}TCR^{+}Thy1.1^{+} thymocytes (Treg). Dots represent individual mice, bars mean values ± SD. ns, not significant, Mann-Whitney test.

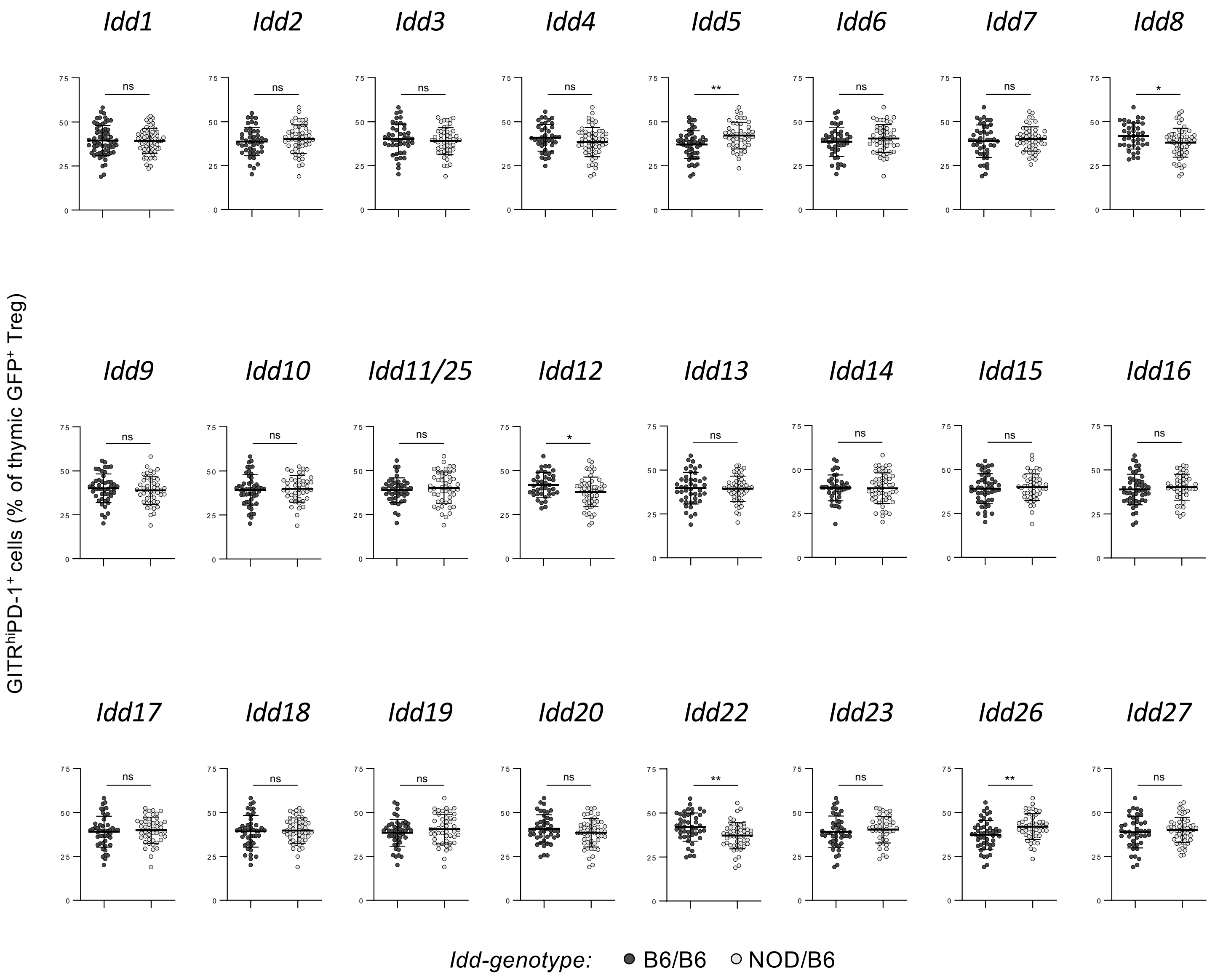

Figure S4. Idd5, 8, 12, 22 and 26, but not other Idd, control development of GITR^{high}PD-1^{+} Treg. Thymocytes from three-day-old (F1xB6)$_{BC1}$ *Rag2-Gfp Foxp3-Thy1^{a}*-mutant neonates were analysed by flow-cytometry, and *Idd*-genotypes were determined by PCR. Indicated are the proportions of GITR^{high}PD-1^{+} cells among GFP^{+}CD4^{+}CD8^{-}TCR^{+}Thy1.1^{+} thymocytes (Treg) in mice carrying indicated *Idd*-genotypes. Dots represent individual mice, bars mean values ± SD. ns, not significant; *, $p<0.05$; **, $p<0.01$, Mann-Whitney test. The data for *Idd5, 8, 12, 22 et 26* are the same as those shown in Fig. 3b.
